# Pulse pressure after thrombectomy predicts functional outcomes and mortality in acute ischemic stroke with large artery occlusion

**DOI:** 10.1038/s41598-025-12962-z

**Published:** 2025-08-12

**Authors:** Shandong Jiang, Zhongju Tan, Jianru Li, Peizheng Guo, Yuan Yuan, Jun Yu, Liang Xu, Xu Li, Xianyi Chen, Bing Fang, Lingling Yu, Jing Xu, Cong Qian, Chaohui Jing, Yunmei Yang

**Affiliations:** 1 Department of Neurological Surgery, The Second Affiliated Hospital of Zhejiang University School of Medicine, Hangzhou, 310003 China; 2 Department of Geriatrics, The First Affiliated Hospital of Zhejiang University School of Medicine, Hangzhou, 310003 China; 3 Department of Nursing, The First People’s Hospital of Xiaoshan District, Hangzhou, 310003 China; 4 Department of Neurological Surgery, Xinhua Hospital Affiliated to Shanghai Jiao Tong University, Shanghai, 200000 China

**Keywords:** Pulse pressure, Ischemic stroke, Endovascular thrombectomy, Blood pressure, Large vessel occlusions, Prognosis, Diseases of the nervous system, Cerebrovascular disorders, Stroke

## Abstract

Post-procedure blood pressure (BP) strongly influences the prognosis of ischemic stroke patients with large vessel occlusion. However, no consensus or guidelines currently exist regarding BP targets and management after endovascular thrombectomy (EVT). Although several recent clinical trials have investigated BP management after EVT, their conclusions were inconsistent and contradictory. In this study, we systematically analyzed 12 post-procedure BP parameters from 826 acute ischemic stroke patients within 24 h after EVT; 587 cases were included in the final analysis. We utilized multivariate logistic regression to identify predictors of poor prognosis and mortality after EVT. Restrictive cubic splines were used to evaluate dose–response relationships of mean pulse pressure (PP) with clinical outcomes. Subgroup analyses were conducted to assess the predictive performance of mean PP across patient subgroups. Mean PP demonstrated statistically significant positive dose–response relationships with poor functional outcomes, symptomatic intracranial hemorrhage (sICH), and mortality after EVT. Elevated mean PP (> 57.39 mmHg) was a stronger predictor of adverse outcomes relative to systolic or diastolic BP alone; it exhibited the highest adjusted odds ratio (aORs 2.39; 95% confidence interval [CI] 1.58–3.62) and area under the curve (AUC = 0.661; 95% CI 0.617–0.705). Mean PP exhibited linear relationships with all other outcome events except mortality at 12 months after EVT. Mean PP within 24 h after EVT is an independent risk factor for sICH, poor functional outcomes, and mortality in stroke patients. BP management strategies should prioritize reducing systolic BP and stabilizing PP fluctuations.

**Supplementary Information:**

The online version contains supplementary material available at 10.1038/s41598-025-12962-z.

## Introduction

Large vessel occlusion (LVO) causes approximately one-third of acute ischemic strokes (AISs) but contributes to more than half of all stroke-related disabilities and deaths^1^. Since 2016, endovascular thrombectomy (EVT) has been an established treatment for patients with LVO-induced AIS^2^. Despite successful recanalization (extended Thrombolysis in Cerebral Infarction [eTICI] score 2B or 3) at the end of the procedure, nearly half of these patients either die or are left severely and permanently disabled (modified Rankin Scale [mRS] 4–6)^3,4^. Recent evidence suggests that hemodynamic management plays a critical role in post-EVT care^5^. Up to 80% of AIS patients experience elevated blood pressure (BP) after the procedure, which may require several days to return to baseline levels^6^. During ischemia, the brain tissue in the infarcted area loses its ability to regulate blood flow, amplifying the effect of systemic BP on intracranial pressure and perfusion^7^. Post-EVT increases and variability in BP have been linked to poor functional outcomes^8^, cerebral edema, and symptomatic hemorrhagic transformation^9^. Although several recent clinical trials have examined BP management following EVT, their findings were inconsistent. Controversies persist regarding whether BP reduction to < 140 mmHg improves prognosis^10^, reduces the incidence of spontaneous cerebral hemorrhage^11^, or indicates the optimal timing for initiation of intensive BP-lowering therapy^12^.

Studies thus far have primarily focused on the relationships of postoperative systolic BP (SBP) with clinical outcomes. However, Lee et al. reported that pulse pressure (PP) might serve as a better prognostic indicator than SBP or mean arterial pressure (MAP) in stroke patients undergoing intravenous thrombolysis (IVT)^13^. PP, an important hemodynamic parameter, reflects the difference between the maximum and minimum pressure values in a cardiac cycle^14^. In previous studies, higher PP at admission has been strongly associated with both the occurrence and recurrence of AIS^15^. For AIS patients undergoing IVT, elevated PP is an independent predictor of poor early outcomes at hospital discharge and increased 30-day mortality risk^16^. Additionally, PP is closely linked to the risk of AIS occurrence^17^ and recurrence^18^. Patients with PP in the highest quartile (> 74 mmHg) exhibit a significantly higher risk of recurrence relative to those with PP in the lowest quartile (< 50 mmHg) (hazard ratio [HR] = 1.56, 95% confidence interval [CI] 1.13–2.15)^17^. Despite these findings, it remains unclear whether the results observed concerning IVT can be directly extrapolated to patients undergoing EVT, considering that few studies have focused on PP in AIS patients after EVT. We speculate that the elevated PP after EVT may affect the homeostasis of hemodynamics, thereby increasing the risk of cerebral hemorrhage transformation in stroke patients with AIS-LVO and thus influencing their prognosis at 3 and 12 months. Therefore, in this study, we analyzed the relationships of post-procedural BP parameters during the first 24 h with primary and secondary outcomes after thrombectomy.

## Methods

### Study design and populations

**Fig. 1:**
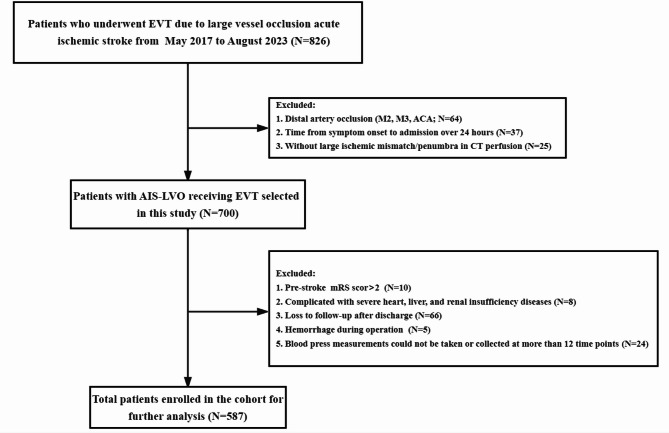
Flow chart of enrolled patients. EVT, endovascular thrombectomy; ICA, internal carotid artery; IVT, intravenous thrombolysis; ACA, anterior communicating artery; M2/M3, second/third segment of the middle cerebral artery; mRS, modified Rankin Scale.

This retrospective study analyzed consecutive AIS patients with LVO who underwent EVT between March 2016 and August 2023 at the Stroke Center of the Second Affiliated Hospital, Zhejiang University (Fig. 1). As a research involving human participants, all methods were performed in accordance with the Declaration of Helsinki. Patients were included based on the following criteria: (1) they underwent thrombectomy using second-generation stent-retriever devices or aspiration systems (e.g., Solitaire AB/FR [Covidien/ev3, Irvine, CA, USA]; Trevo Proview, Stryker, [Fremont, CA, USA]); (2) they exhibited digital subtraction angiography (DSA)-confirmed LVO, including the internal carotid artery (ICA), middle cerebral artery (MCA M1/M2), basilar artery (BA), or vertebral artery (VERT); (3) they displayed an mRS score ≤ 1 before stroke onset; (4) they had undergone EVT within 6 h of symptom onset or within 6–24 h in the presence of a large ischemic mismatch/penumbra, as determined by computed tomography (CT) perfusion^19^.

Exclusion criteria were as follows: (1) presence of intracranial hemorrhage on CT scan prior to EVT; (2) severe cardiac insufficiency (ejection fraction < 40%), hepatic dysfunction (prothrombin activity [PTA] ≤ 40%), renal insufficiency (serum creatinine > 707 μmol/L), or advanced diabetes with blood glucose levels exceeding 22 mmol/L^20^; (3) absence of > 40% of postoperative BP records (> 10 time points) due to postoperative examinations or a second operation to manage complications^21^; (4) lack of follow-up imaging examinations or mRS scores at 3 months after EVT. This rigorous selection process ensured a homogeneous study population representative of clinical practice while minimizing confounding factors that could influence study outcomes.

### Demographic, clinical and comorbidities data

Data for our study were extracted from the electronic medical records system, which is supported by the Stroke Center at the Department of the Second Affiliated Hospital, Zhejiang University. The patients included in our study underwent endovascular thrombectomy (EVT), encompassing a range of procedures such as thrombectomy with stent retrievers, thrombus aspiration, intracranial angioplasty, stent implantation, or any combination thereof, as determined by the treating surgeon. The occlusion sites of the arteries were identified through computed tomography angiography (CTA), magnetic resonance angiography (MRA), and/or cerebral digital subtraction angiography (DSA) reports, and included vessels such as the internal carotid artery (ICA), middle cerebral artery (MCA), basilar artery (BA), and vertebral artery (VERT).

The collected data were categorized into several domains, including baseline demographic data, medical history, stroke characteristics, procedural variables, treatment variables, and hospitalization complications, totaling 49 variables. Baseline characteristics encompassed age, sex, National Institutes of Health Stroke Scale (NIHSS) score, and modified Rankin Scale (mRS) score at admission, as well as comorbidities such as diabetes, hypertension, atrial fibrillation, hyperlipidemia, and a history of stroke, along with the use of antiplatelet and anticoagulation medications. Procedural time variables included metrics such as time from puncture to reperfusion (TPR), time from onset to groin puncture (OTP), time from onset to reperfusion (OTR), time from onset to admission (OTA), time from onset to imaging (OTI), and time from imaging to puncture (ITP). Additionally, we recorded the use of tissue plasminogen activator (t-PA), the number of retrieval attempts exceeding three, and rescue therapies including balloon angioplasty and stenting. Reperfusion status was assessed using the modified Thrombolysis in Cerebral Infarction (mTICI) scale by surgeons during the operation, with mTICI grades better than 2B indicating successful recanalization. Hospitalization complications such as hematoma transformation, symptomatic intracranial hemorrhage (sICH), respiratory failure, liver dysfunction, and pulmonary infection were also documented.

### BP monitoring, measurement, and collection

We collected SBP and diastolic blood pressure (DBP) records from the end of the EVT procedure (defined as the time of reperfusion or the last contrast bolus) until 24 h post-EVT^10^. After EVT, SBP and DBP were measured at 15-min intervals during the first 2 h, 30-min intervals from 3 to 6 h, and 1-h intervals from 7 to 24 h^10^. Twenty-four patients were excluded from the study because they had fewer than 12 recorded BP measurements within the first 24 h after EVT. Among the 572 patients included in the final analysis, none had missing BP records; all had complete BP data for the first 12 h. Anti-hypertensive medication selection followed AIS treatment guidelines; such medication was given at the discretion of the treating physicians, and calcium channel blockers were most commonly prescribed. Additionally, a sensitivity analysis was performed to evaluate the associations of predefined BP measures during the first 24 h after EVT with clinical outcomes.

### Follow-up and outcomes

Patients were dichotomized by favorable or unfavorable outcomes groups based on mRS score at 3 months after EVT were 0–3 or 4–6. The follow-up protocol was consistent with previously published literatures^22^. The primary outcome measure was functional outcome according to the modified Rankin Scale, which is a 7-point scale ranging from 0 (no symptoms) to 6 (death), assessed at 90 days after EVT^22^. The scores were collected by a stroke neurologist during routine follow-up visits at 90 days (± 14) after stroke for the majority of patients through telephone discussion or clinical follow-up with patients or their families. Secondary outcome was defined as mortality at discharge, 3 months and 12 months and sICH. Hemorrhagic transformation (HT) referred to the bleeding caused by the reperfusion of blood vessels in the ischemic area after acute cerebral infarction^23^. sICH was defined as any intracranial hemorrhage with an increase in the NIHSS score of ≥ 4 from baseline) within 7 days after EVT, according to the ECASS (European-­Australasian Acute Stroke Study) II criteria^23^.

### Statistical analysis

Details of missing data are provided in Online supplementary figure 1. For variable data (OTP and BNP) with missing more than 20%, we directly exclude them to minimize the bias resulting from missing data. For variables with missing less than 5%, the median is used to simply interpolate and fill in the data. For the variables with a missing ratio ranging from 5 to 20%, we used fivefold multiple interpolation for filling and Trace plot for diagnosis and evaluation. Characteristics of the study population were summarized as proportions for categorical variables and as mean ± standard deviation (SD) or median (25th–75th percentile) for continuous variables, as appropriate. Two-sample t-tests or the Mann–Whitney U test were used to compare continuous quantitative variables, whereas χ2 tests or Fisher’s exact test were utilized to compare categorical variables. Analysis of variance (ANOVA) and univariate logistic regression identified nine potential BP parameters significantly associated with primary and secondary outcomes in AIS patients (*P* < 0.05). To elucidate the effects of postoperative BP parameters on prognosis, progressive forward logistic regression was conducted to adjust for potential confounding factors. Covariates included sex, advanced age (≥ 80 years), successful recanalization (eTICI score 2B or 3), general anesthesia, National Institutes of Health Stroke Scale (NIHSS) score > 14, hypertension, atherosclerosis, coronary heart disease (CHD), diabetes, occlusion site, atrial fibrillation, and more than three thrombectomy passes. After adjustment for these confounding factors, four variables (mean SBP, mean PP, maximum SBP, and SBP-DMM) were identified as independent predictors of short- and long-term prognosis after EVT. After correction for the combined effects of the four variables, mean PP emerged as the sole independent risk factor and predictor of poor prognosis. All variance inflation factors (VIFs) for the model were < 10, indicating no multicollinearity issues. Results are presented as adjusted odds ratios (aORs) with corresponding 95% confidence intervals (CIs). The dose–response relationship between mean PP during hospitalization and adverse outcomes of ischemic stroke was evaluated using a restricted cubic spline (RCS) model with four nodes. The optimal cut-off value for mean PP was determined based on the Akaike Information Criterion (AIC) in the RCS model. Receiver operating characteristic (ROC) analysis was performed to identify the cut-off values of predictors and assess their accuracy in short- to medium-term prognosis prediction. Subgroup analyses were conducted to verify the robustness of the findings and identify potential interaction factors. *P* values < 0.05 were considered statistically significant, and all reported P-values were two-sided. Statistical analyses were performed using IBM SPSS Statistics for Windows version 26 and R statistical software version 4.0.5.

## Results

### Baseline characters of cohort

Among 587 patients finally enrolled in the cohort, 326 (55.54%) had an unfavorable 3-months prognosis after EVT who were unable to walk independently. The characteristics of the patients are shown in Table 1. Among the cohort, the patients’ mean ± SD age was 69.02 ± 13.30 years, with 333 (56.73%) being male and a median admission mRS score of 4. Patients with poor prognosis had a significantly higher mean age, time from puncture to reperfusion (PTR), baseline NHISS and mRS score on admission and with higher proportion of female, hypertension, CHD, diabetes, atrial fibrillation, general anesthesia (unable to cooperate with agitation), difficult thrombectomy (greater than three times). Conversely, higher proportion of successful reperfusion (eTICI = 2B or 3) and MCA occlusion but lower proportion of VERT/BA occlusion was observed in patients with favorable prognosis. Then, above significant changing variables were adjusted for analysis of the association between BP variables and primary and secondary outcome after EVT.

**Table 1:** Baseline characteristics and blood pressure parameters of patients with favorable or unfavorable outcomes after 3 months for endovascular treatment of acute large artery occlusive ischemic stroke.

Variables	Total patients (n = 587)	Favorable prognosis (n = 261)	Unfavorable prognosis (n = 326)	*P*
Age, Mean ± SD	69.02 ± 13.30	64.69 ± 13.45	72.48 ± 12.13	**< 0.001**
BMI, Mean ± SD	23.40 ± 3.42	23.66 ± 3.34	23.19 ± 3.46	0.099
Male, n (%)	333 (56.73)	160 (61.30)	173 (53.07)	**0.045**
Age higher than 80 years, n (%)	132 (22.49)	27 (10.34)	105 (32.21)	**< 0.001**
Procedural variables				**0.924**
OTA, Mean ± SD	311.28 ± 219.38	305.06 ± 200.81	316.33 ± 233.58	0.546
OTI, Mean ± SD	272.48 ± 229.76	263.38 ± 217.23	279.82 ± 239.47	0.397
ITP, Mean ± SD	118.76 ± 96.91	110.08 ± 59.63	125.73 ± 118.31	0.062
PTR, Mean ± SD	82.32 ± 66.30	73.74 ± 42.81	89.21 ± 79.75	**0.007**
OTR, Mean ± SD	452.13 ± 229.74	434.95 ± 216.40	465.93 ± 239.39	0.119
OTP, Mean ± SD	370.20 ± 218.37	361.75 ± 210.97	376.98 ± 224.25	0.421
Operation history, n (%)	196 (33.39)	83 (31.80)	113 (34.66)	0.465
Tirofiban treatment, n (%)	103 (17.55)	52 (19.92)	51 (15.64)	0.176
Stent pass attempts > 3, n (%)	92 (15.67)	32 (12.26)	60 (18.40)	**0.042**
Medical history				
CIA, n (%)	130 (22.15)	52 (19.92)	78 (23.93)	0.246
Hyperlipemia, n (%)	22 (3.75)	10 (3.83)	12 (3.68)	0.924
Hypertension, n (%)	378 (64.40)	145 (55.56)	233 (71.47)	**< 0.001**
CHD, n (%)	69 (11.75)	22 (8.43)	47 (14.42)	**0.025**
Diabetes, n (%)	110 (18.74)	29 (11.11)	81 (24.85)	**< 0.001**
Atrial fibrillation, n (%)	219 (37.31)	82 (31.42)	137 (42.02)	**0.008**
Pre-antiplatelet, n (%)	70 (11.93)	35 (13.41)	35 (10.74)	0.321
Pre-anticoagulation, n (%)	81 (13.80)	34 (13.03)	47 (14.42)	0.627
Treatment variable				
Rescue therapy^#^, n (%)	105 (17.89)	46 (17.62)	59 (18.10)	0.882
Distal escape, n (%)	42 (7.16)	20 (7.66)	22 (6.75)	0.669
IVT, n (%)	344 (58.60)	159 (60.92)	185 (56.75)	0.308
Successful recanalization (mTICI = 2B/3), n (%)	544 (92.67)	250 (95.79)	294 (90.18)	**0.01**
General anesthesia, n (%)	303 (51.62)	120 (45.98)	183 (56.13)	**0.014**
Stroke characteristics				
mRS at admission, M (Q_1_, Q_3_)	4.00 (3.00, 5.00)	3.00 (2.00, 4.00)	5.00 (5.00, 5.00)	**< 0.001**
NHISS > 14, n (%)	384 (65.42)	140 (53.64)	244 (74.85)	**< 0.001**
Multistage thrombus, n (%)	98 (16.70)	39 (14.94)	59 (18.10)	0.308
TOAST classification, n (%)				0.524
LAA	173 (45.77)	94 (47.72)	79 (43.65)	
CE	179 (47.35)	88 (44.67)	91 (50.28)	
Others or undetermined etiology	26 (6.88)	15 (7.61)	11 (6.08)	
Site of occlusion, n (%)				**< 0.001**
ICA	104 (17.72)	43 (16.48)	61 (18.71)	
MCA	313 (53.32)	162 (62.07)	151 (46.32)	
ICA-MCA	58 (9.88)	22 (8.43)	36 (11.04)	
BA/VA	76 (12.95)	17 (6.51)	59 (18.10)	
ACOA/PCoA	36 (6.13)	17 (6.51)	19 (5.83)	
Hospitalization complications				
HT, n (%)	191 (32.54)	48 (18.39)	143 (43.87)	**< 0.001**
sICH, n (%)	120 (20.44)	3 (1.15)	117 (35.89)	**< 0.001**
Pulmonary infection, n (%)	276 (47.02)	99 (37.93)	177 (54.29)	**< 0.001**
Liver dysfunction, n (%)	73 (12.44)	30 (11.49)	43 (13.19)	0.536
Respiratory failure, n (%)	53 (9.03)	7 (2.68)	46 (14.11)	**< 0.001**
Blood pressure variables				
Baseline SBP, M (Q_1_, Q_3_)	145.00 (128.00, 162.00)	142.00 (124.00, 159.00)	148.00 (130.00, 165.00)	**0.009**
Baseline DBP, M (Q_1_, Q_3_)	81.00 (72.00, 91.00)	80.00 (72.00, 90.00)	82.00 (71.00, 92.00)	0.672
Baseline PP, M (Q_1_, Q_3_)	63.00 (49.00, 78.00)	60.00 (46.00, 75.00)	64.50 (52.00, 79.75)	**0.003**
Mean SBP, M (Q_1_, Q_3_)	126.92 (118.58, 135.02)	124.17 (115.83, 133.50)	128.77 (121.36, 136.30)	**< 0.001**
Mean DBP, M (Q_1_, Q_3_)	68.04 (62.62, 74.15)	68.75 (63.33, 75.62)	67.82 (62.04, 72.65)	**0.013**
Mean PP, M (Q_1_, Q_3_)	57.45 (49.60, 66.50)	53.08 (46.88, 61.29)	61.12 (53.22, 69.43)	**< 0.001**
Maximum SBP, M (Q_1_, Q_3_)	155.00 (144.00, 166.00)	151.00 (139.00, 161.00)	158.00 (150.00, 169.00)	**< 0.001**
Minimun SBP, M (Q_1_, Q_3_)	103.00 (96.00, 111.00)	102.00 (95.00, 110.00)	104.00 (96.00, 113.00)	0.202
Maximum DBP, M (Q_1_, Q_3_)	88.00 (81.00, 98.00)	88.00 (81.00, 98.00)	88.00 (80.00, 98.00)	0.804
Minimun DBP, M (Q_1_, Q_3_)	53.00 (47.00, 58.00)	53.00 (49.00, 60.00)	52.00 (46.00, 57.00)	**0.002**
SBP-DMM, M (Q_1_, Q_3_)	50.00 (40.00, 62.00)	46.00 (37.00, 58.00)	53.00 (43.00, 65.00)	**< 0.001**
DBP-DMM, M (Q_1_, Q_3_)	34.00 (29.00, 42.50)	33.00 (28.00, 41.00)	35.00 (29.00, 43.75)	**0.024**

CHD, Coronary heart disease; MRS, Modified Rankin Scale; ICA, Internal carotid artery; MCA, Middle cerebral artery, PCoA, posterior communicating artery; ACoA, anterior communicating artery; VA, vertebral artery; BA, basilar artery; NIHSS, National Institutes of Health Stroke Scale; TPR, time from puncture to reperfusion; OTP, time from onset to groin puncture; OTR, time from onset to reperfusion; OTA, time from Onset to admission; OTI, time from Onset to image; ITP, time from image to puncture; LAA, large artery atherosclerosis; CE, cardioembolism; IVT, intravenous thrombolysis; mTICI, modified Thrombolysis in Cerebral Infarction; HT, hemorrhagic transformation; sICH, symptomatic intracranial hemorrhage; SBP, systolic blood pressure; DBP, diastolic blood pressure; PP, pulse pressure; DMM, difference between maximum and minimum.

Qualitative variable are n (%); quantitative variables are mean ± SD or M (Q_1_, Q_3_). SD: standard deviation, M: Median, Q_1_: 1st Quartile, Q_3_: 3st Quartile. *P < 0.05; **P < 0.001.

^#^Rescue therapy included balloon angioplasty and permanent stenting.

Significant values are in bold.

Nine BP parameters were significantly different between two different outcomes in Online supplementary Table 1. Post-procedural SBP parameters (including baseline SBP, mean SBP, maximum SBP, and SBP-DMM) were significantly higher in patients with poor functional outcomes than in those with good functional outcomes. Inversely, patients with unfavorable prognosis exhibited lower post-procedural DBP parameters (including mean DBP, minimum DBP, and DBP-DMM) than patients with favorable prognosis. As a parameter reflecting dynamic changes in BP, patients with unfavorable prognosis presented higher mean PP.

### Association of Blood pressure variables with primary and secondary outcomes after EVT

We have adjusted for eleven confounding factors with a significant difference of *P* < 0.05 in Table 2 and online supplementary Table 2. The trend curves of SBP, DBP and PP changes within 24 h after EVT for patients with good prognosis and those with poor prognosis was depicted in online Fig. 3. In short-term prognosis, mean SBP (aOR 1.02; 95% CI 1.01–1.04), maximum SBP (aOR 1.02; 95% CI 1.01–1.03), SBP-DMM (aOR 1.02; 95% CI 1.01–1.03), and mean PP (aOR 1.04; 95% CI 1.02–1.06) within 24 h post-procedural were significantly associated with unfavorable functional outcomes. In long-term prognosis, in addition to the above four variables, DBP-DMM (aOR 1.02; 95% CI 1.01–1.04), and minimum DBP (aOR 0.98; 95% CI 0.96–0.99) were significantly associated with unfavorable functional outcomes. Mean SBP, mean PP, maximum SBP and SBP-DMM are both independently associated with functional outcomes at 3 and 12 months, in which the correlation of mean PP to prognosis was the strongest with the highest aORs. Then, we further explored the association between above four variables and mortality (at discharge, 3 months and 12 months) and sICH in online supplementary Table 3. We found that mean PP was the independent risk factor and predictor of mortality at admission (aOR 1.07; 95% CI 1.02–1.12), mortality at 3 months (aOR 1.02; 95% CI 1.01–1.04), mortality at 12 months (aOR 1.03; 95% CI 1.01–1.04) and sICH (aOR 1.02; 95% CI 1.01–1.04) with the highest ORs than other three variables. Sensitive analysis on the original data that were not excluded patients with missing data also indicated significant relationship between mean PP and prognosis and death of patients after EVT in online supplementary Table 6.

**Table 2:** Unadjust and adjusted ORs of the association of post-procedure blood pressure variables with unfavorable functional prognosis at 3 and 12 months after endovascular treatment.

Blood pressure variables	Unfavorable functional prognosis at 3 months					Unfavorable functional prognosis at 12 months				
	Univariate		Multivariate			Univariate		Multivariate		
	OR (95% CI)	*P*	aβ	aOR (95% CI)	*aP*	OR (95% CI)	*P*	aβ	aOR (95% CI)	*aP*
Mean SBP	1.03 (1.02–1.05)	**< 0.001**	0.02	1.02 (1.01–1.04)	**0.005**	1.03 (1.02–1.05)	**< 0.001**	0.02	1.02 (1.01–1.04)	**0.011**
Mean DBP	0.97 (0.96–0.99)	**0.005**	− 0.02	0.98 (0.96–1.00)	0.08	0.98 (0.96–0.99)	**0.017**	− 0.01	0.99 (0.96–1.01)	0.182
Mean PP	1.05 (1.03–1.07)	**< 0.001**	0.04	1.04 (1.02–1.06)	**< 0.001**	1.05 (1.03–1.06)	**< 0.001**	0.03	1.03 (1.01–1.05)	**< 0.001**
Maximum SBP	1.03 (1.02–1.04)	**< 0.001**	0.02	1.02 (1.01–1.03)	**< 0.001**	1.03 (1.02–1.04)	**< 0.001**	0.02	1.02 (1.01–1.04)	**< 0.001**
Maximum DBP	1.00 (0.98–1.01)	0.549	**–**	1.00 (0.99–1.01)	0.968		**–**			
Minimum SBP	1.00 (0.99–1.02)	0.462	**–**	1.00 (0.99–1.01)	0.885		**–**			
Minimum DBP	0.96 (0.95–0.98)	**< 0.001**	-0.03	0.97 (0.95–1.00)	0.075	0.97 (0.95–0.99)	**0.002**	− 0.02	0.98 (0.96–0.99)	**0.041**
SBP-DMM	1.02 (1.01–1.03)	**< 0.001**	0.02	1.02 (1.01–1.03)	**< 0.001**	1.03 (1.02–1.04)	**< 0.001**	0.02	1.02 (1.01–1.04)	**< 0.001**
DBP-DMM	1.02 (1.01–1.03)	**0.027**	0.01	1.01 (1.00–1.03)	0.105	1.02 (1.01–1.03)	**0.013**	0.02	1.02 (1.01–1.03)	**0.04**

Adjusting for confounders included the baseline and procedural variables with a significant difference of *P* < 0.05 (male, age higher than 80 years, successful recanalization (mTICI = 2B or 3), genernal anesthesia, NHISS higher than 14, hypertension, CHD, diabetes, site of occlusion, atrial fibrillation and number of pass time over 3).

Abbreviation: SBP, systolic blood pressure; DBP, diastolic blood pressure; PP, pulse pressure; DMM, difference between maximum and minimum.

OR: Odds Ratio, CI: Confidence Interval. *P < 0.05; **P < 0.001.

Significant values are in bold.

### Mean PP may be a better predictor of outcomes after EVT than SBP

We used the receiver operating characteristic curve (ROC) to further validate the diagnostic efficacy of the above four variables in online Fig. 2. Among them, mean PP within 24 h post-procedure had the optimal diagnostic efficacy on poor outcome after EVT with the cut-off value of 57.39 mmHg, presenting a sensitivity of 72.7% and a specificity of 65.1% with the AUC of 0.661 (95% CI 0.617–0.705). When we corrected the above four risk factors at the same time, mean PP was the only independent risk factor and predictor of poor prognosis at 3 months (aOR 1.04; 95% CI 1.01–1.07) and 12 months (aOR 1.03; 95% CI 1.01–1.06), mortality at discharge (aOR 1.09; 95% CI 1.02–1.17), and mortality at 3 months (aOR 1.04; 95% CI 1.01–1.06) in online supplementary Table 4 and 5.

**Fig. 2:**
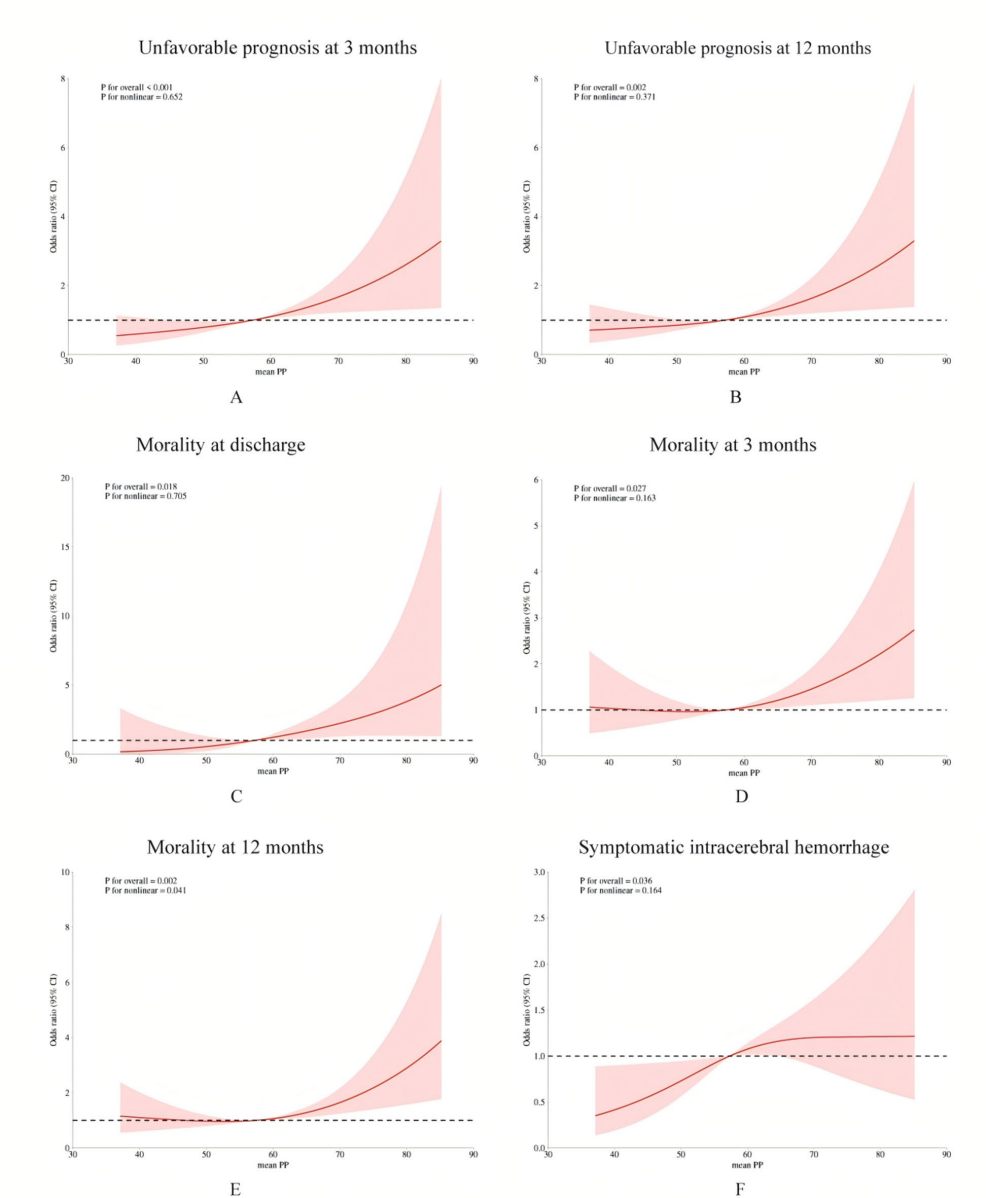
(**A**) The dose–response relationship between postoperative mean PP and unfavorable prognosis at 3 months through restricted cubic splines (RCS) with 3 knots. (**B**) The dose–response relationship between postoperative mean PP and unfavorable prognosis at 12 months. (**C**) The dose–response relationship between postoperative mean PP and mortality at discharge. (**D**) The dose–response relationship between postoperative mean PP and mortality at 3 months. (**E**) The dose–response relationship between postoperative mean PP and mortality at 12 months. (**F**) The dose–response relationship between postoperative mean PP and symptomatic intracerebral hemorrhage.

Restricted cubic spline (RCS) with 3 knots was used to explore the relationship between postoperative PP and outcome variables after adjusting confounding factors in Fig. 2. Mean PP demonstrates a significant dose–response relationship with the occurrence of functional outcomes, sICH, and mortality after thrombectomy, with an increased risk of adverse outcomes as PP rises. Besides, mean PP exhibits a linear relationship with all other outcome events, except for mortality at 12 months post-EVT.

### Association of elevated mean PP with primary and secondary outcomes after EVT

Based on the cut-off value determined by RCS, the continuous mean PP variable was categorized into groups above 57.39 mmHg and below 57.39 mmHg. Compared with patients with lower mean PP (< 57.39 mmHg), patients with higher mean PP within in 24 h following EVT were more likely to complicated sICH (aOR 1.06; 95% CI 1.01–2.55) and have worse functional outcomes at 3 months and 12 months (aOR 2.39; 95% CI 1.58–3.62; aOR 2.08; 95% CI 1.37–3.14) or higher risk of mortality at discharge (aOR 8.00; 95% CI 1.68–38.12) and at 12 months after EVT (aOR 1.66; 95% CI 1.1–2.50) in Table 3.

**Table 3:** Adjusted ORs of the association of elevated mean PP (> 57.39 mmHg) post-procedure with primary and second outcome events after endovascular treatment.

Outcome events	Univariate		Multivariate^a^		
	OR (95% CI)	*P*	aβ	aOR (95% CI)	*aP*
Primary outcome					
Unfavorable prognosis at 3 months	3.08 (2.20–4.33)	< 0.001	0.87	2.39 (1.58–3.62)	< 0.001
Unfavorable prognosis at 12 months	2.76 (1.97–3.86)	< 0.001	0.73	2.08 (1.37–3.14)	< 0.001
Second outcome					
Mortality at discharge	9.49 (2.18–41.27)	**0.003**	2.08	8.00 (1.68–38.12)	**0.009**
Mortality at 3 months	1.88 (1.29–2.75)	**0.001**	0.42	1.52 (0.99–2.33)	0.058
Mortality at 12 months	1.99 (1.40–2.82	** < 0.001**	0.51	1.66 (1.11–2.50)	**0.014**
Symptomatic intracerebral hemorrhage	1.97 (1.30–2.98)	**0.001**	0.47	1.60 (1.01–2.55)	**0.048**

^a^Adjust variables of outcome after 3 months incorporate PTR, male, age higher than 80 years, successful recanalization (mTICI ≥ 2B/3), genernal anesthesia, HT, sICH, pulmonary infection, respiratory failure, NHISS higher than 14, hypertension, CHD, diabetes, site of occlusion, atrial fibrillation and number of sent pass over three times.

SBP, systolic blood pressure; DBP, diastolic blood pressure; PP, pulse pressure; PTR, time from puncture to reperfusion.

OR: Odds Ratio, CI: Confidence Interval. *P < 0.05; **P < 0.001.

Significant values are in bold.

### Subgroup analysis of post-procedure PP with functional outcomes

**Fig. 3:**
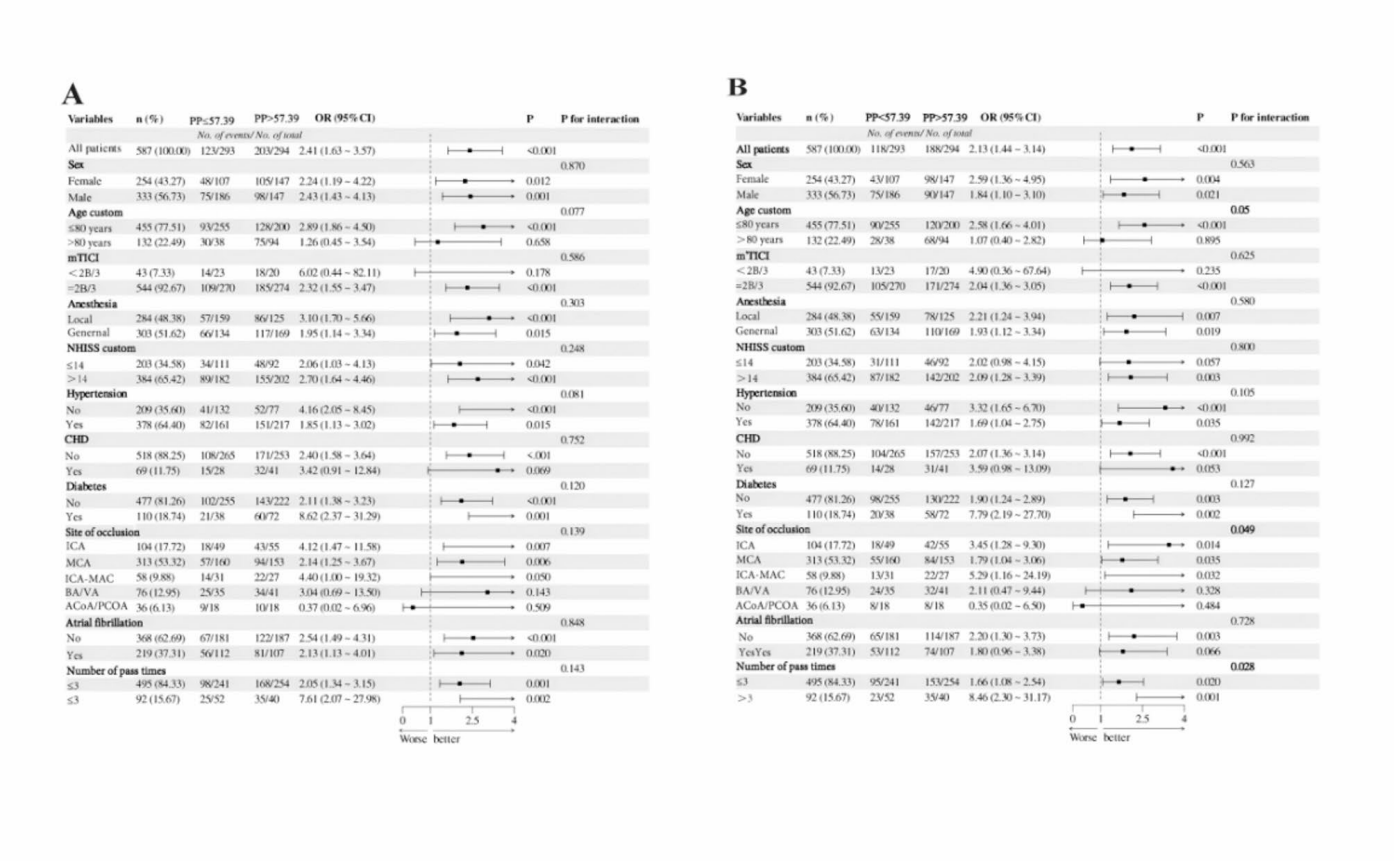
(**A**) Subgroup analysis of patients with 90-day modified Rankin Scale (mRS) score of 0–3. (**B**) Subgroup analysis of patients with 1-year modified Rankin Scale (mRS) score of 0–3.

Subgroup variables were selected from positive clinical risk factors after multivariate correction. In most subgroups, higher PP group (> 57.39 mmHg) was significantly associated with poor functional outcomes at 3 and 12 months after EVT in Fig. 3. Regardless of patients’ gender, type of anesthesia, presence or absence of comorbidities such as hypertension and diabetes, and whether the number of thrombectomies exceeds three times, controlling PP below 57.39 mmHg is beneficial for improving prognosis. However, there is no significant interaction effects between PP custom and various stratification factors on outcomes at 3 months post-thrombectomy (all *P* > 0.05). In term of outcome at 12 months, we unexpectedly found that in the subgroup characterized by more than three thrombectomy procedures, age less than 80 years, and anterior circulation infarction, the benefit of maintaining PP below 57.39 mmHg is significantly greater than that of its corresponding subgroup.

## Discussion

Our study demonstrated that mean SBP, mean PP, maximum SBP, and SBP-DMM were all significantly and positively associated with poor functional prognosis at 3 and 12 months. Among these parameters, mean PP showed a linear relationship with most outcome events in the RCS analysis, except for mortality at 12 months post-EVT. The predictive power and strength of the association between mean PP and prognosis were superior to those of single SBP or DBP, as evidenced by the strongest aORs and highest diagnostic performance (AUC = 0.661, 95% CI 0.617–0.705). Mean PP in the first 24 h was closely associated with both primary and secondary outcomes. It was an independent risk factor and predictor of poor prognosis, as well as an independent risk factor for mortality and sICH after EVT. Subgroup analysis revealed that mean PP maintenance below 57.39 mmHg was significantly associated with favorable prognosis after EVT in several specific groups, including those who underwent more than three thrombectomy procedures, were aged < 80 years, and experienced anterior circulation infarctions. However, missing post-EVT BP data and incomplete follow-up records for some patients led to the exclusion of valuable cases, potentially introducing selection and survival biases. We sought to eliminate systematic errors by conducting a sensitivity analysis of the original dataset (including patients with missing data); this analysis explored the relationships of mean PP with patient prognosis and mortality after thrombectomy. The results remained statistically significant, as shown in Online supplementary Table 6, indicating that the missing data were missing at random. We also conducted a baseline difference analysis of the included and excluded patients to compare the baseline equilibrium in Online supplementary Table 7. The results showed that there was no significant difference in all the variables between included and excluded patients, which may collectively mitigate concerns about selection bias influencing our conclusions.

High PP, determined by the interplay of cardiac stroke volume, the systemic arterial system, and peripheral microcirculatory pressure, may reflect impaired BP autoregulatory function^24^. PP is primarily influenced by poor large vessel compliance and is closely associated with atherosclerosis severity in these vessels. Postoperative fluctuations and increases in PP may result from large-vessel atherosclerosis. In our study, we adjusted for comorbidities such as CHD, atrial fibrillation, and atherosclerosis. The results remained statistically significant, suggesting that mean PP is an effective predictor of poor prognosis and mortality after thrombectomy, independent of atherosclerosis. We speculate that increases and fluctuations in PP during BP reduction after EVT may have contributed to the neutral or negative findings of previous randomized controlled trials (RCTs) concerning BP management. Although the associations and diagnostic efficiency of PP in this study were superior to those of SBP or DBP alone, PP may function as an indirect marker rather than a uniquely actionable parameter.

When ischemic occur, excessive systemic BP may lead to a breach in cerebral perfusion pressure, resulting in hemorrhagic transformation, while too low systemic pressure may cause inadequate perfusion pressure in a decline in collateral compensation capacity, further exacerbating the infarction^25^. Therefore, balancing the risks of intracranial hyperperfusion and hypoperfusion by optimal post-procedure peripheral BP and perfusion pressure is an important part of the treatment during hospitalization for patients with AIS^26^. American Heart Association/American Stroke Association (AHA/ASA) guidelines recommend a fixed blood pressure target of ≤ 180/105 mmHg during and for 24 h after the procedure (IIa recommendation)^19^. However, the above recommendations are mainly based on the relevant evidence of IVT. At present, there is limited data to guide BP management for AIS-LVO patients receiving EVT, and no unequivocal consensus exists regarding the optimal BP target before, during, and after the EVT procedure. Despite recently completed several clinical trials on BP managements post-EVT, the conclusions remain inconsistent. Safety and efficacy of intensive blood pressure lowering after successful endovascular therapy in acute ischemic stroke (BP-TARGET) was the first RCT to investigate BP management after mechanical thrombectomy, which showed that intensive BP lowering group (100 to 129 mmHg) did not significantly reduce the risk of spontaneous hemorrhagic transformation than control group (130 to 185 mmHg), and there was no statistically significant difference in clinical prognosis between the two groups (aOR 0.96, 95% CI 0.60 to 1.51, *P* = 0.84)^27^. The OPTIMAL-BP study incorporated a total of 306 participants, which obtained a similar result to the ENCHANTED2/MT^28^. The positive prognosis (mRS = 0 to 2) at 3 months after surgery in the intensive antihypertensive group (SBP < 140 mmHg) was worse than that in the control group (SBP = 140 to 180 mmHg) (39.4% vs 54.4%; aOR 0.56, 95% CI 0.33 to 0.96), which was terminated early due to safety concerns. The ENCHANTED2/MT study is designed to study whether intensive antihypertensive therapy (SBP < 120 mmHg) could improve functional outcomes in patients over higher BP management strategies (SBP = 140 to 180 mmHg) in patients with AIS-LVO who have successfully recanalized after EVT^10^. The large RCT intended to include 2257 participants and was terminated early in the interim analysis due to reaching the safety endpoint ahead, eventually ended with 821 participants, demonstrated for the first time that a hypertensive strategy with a SBP < 120 mmHg was harmful for patients with AIS-LVO after successfully recanalization (aOR 1.37, 95% CI 1.07 to 1.76), verifying the lower limit of safety management^10^. The optimal BP target to avoid both postoperative hemorrhagic transformation and cerebral perfusion injury remains unknown.

In previous studies, it has been reported that higher PP at admission was closely related to the occurrence and recurrence of AIS^15^. A cohort study involving 9,901 patients with hypertension demonstrated that compared to patients in the lowest quartile (< 50 mmHg), those in the highest quartile (> 74 mmHg) had a significantly increased risk of occurrence of AIS (HR: 1.555, 95% CI 1.127–2.146)^15^. Another study incorporating 1009 young ischemic stroke patients indicated that PP also was a risk factor for recurrence of AIS, which demonstrated that patients with higher admission PP had a higher risk of stroke recurrence (HR: 1.11, 95% CI 1.01 to 1.21)^18^. High PP was also an independent risk factor for the development of AIS, with the highest quartile (> 74 mmHg) at a significantly increased risk than the lowest quartile (< 50 mmHg) (HR: 1.56, 95% CI 1.13 to 2.15)^17^. Another study involving 674 patients with AIS treated with IVT found that higher PP and fluctuations were significantly associated with poor neurological function within 24 h and adverse functional outcomes at 90 days, and were independently associated with the risk of death within 90 days (OR: 1.60, 95% CI 1.23–2.07), suggesting that monitoring and management of PP are crucial in the treatment and prognosis evaluation of AIS^29^.

It is unclear whether post-thrombectomy PP influences prognosis. The present study demonstrated that mean PP post-procedure is an independent predictor of outcomes at 3 and 12 months; it is also closely associated with sICH and postoperative mortality. The predictive power of PP and its strength of association with prognosis are superior to the corresponding metrics for SBP or DBP alone; PP exhibits the highest diagnostic performance and aORs. The mechanism by which PP reduction improves stroke prognosis and lowers sICH incidence remains uncertain. One possible explanation is that the ischemic penumbra is highly sensitive to changes and excessive fluctuations in BP after ischemic stroke, which exacerbate local ischemia and lead to poor outcomes^30^. Additionally, PP may be linked to cerebrovascular lesions and ischemia-induced white matter damage, potentially contributing to cognitive decline and other neurological disorders. Older people with elevated PP exhibit a greater risk of neuronal injury, and PP reduction may improve mRS scores by mitigating progressive cerebrovascular injury^31^. In the subgroup characterized by more than three thrombectomy procedures, age under 80 years, and anterior circulation infarction, PP maintenance below approximate 57 mmHg provided substantially greater benefits relative to other subgroups. This finding may help identify the optimal patient population for PP management.

In this study, we found that mean SBP, maximum SBP, and SBP-DMM were associated with AIS prognosis after EVT, consistent with previous studies^8,32^. A multicenter observational study showed that patients with post-procedural SBP targets of < 140 mmHg and < 160 mmHg achieved significantly better functional outcomes relative to those with the guideline-recommended target of < 180 mmHg^32^. Furthermore, a 24-h mean SBP of < 140 mmHg post-EVT was associated with functional independence; higher SBP levels were independently correlated with sICH, mortality, and a need for hemicraniectomy^8^. A meta-analysis of seven RCTs (MR CLEAN, ESCAPE, EXTEND-IA, SWIFT PRIME, REVASCAT, PISTE, and THRACE) revealed that SBP was associated with worse clinical outcomes in patients exhibiting baseline SBP ≥ 140 mmHg (aOR 0.86, 95% CI 0.81–0.91). However, no significant association was observed in patients with baseline SBP < 140 mmHg^33^. High maximum SBP levels after mechanical thrombectomy (MT) are independently associated with an increased likelihood of 3-month mortality and functional dependence among patients with AIS-LVO. Moderate BP control is also associated with lower odds of 3-month mortality relative to permissive hypertension^34^. Considering ENCHANTED2/MT and OPTIMAL-BP have demonstrated the potentially harmful effects of BP lowering during the acute phase in stroke patients, our result support our speculation that mean PP within 24 h after EVT is an independent risk factor for sICH, poor functional outcomes, and mortality in stroke patients. Clinically, there are various types of antihypertensive drugs available for selection, such as β-blockers, calcium channel blockers, diuretics, ACEIs and etc. When using antihypertensive drugs to control SBP within the target range, it may also have a significant impact on DBP, thereby causing large fluctuations in PP. We can achieve the goal of lowering PP by choosing drugs that reduce SBP but have a relatively small impact on DBP. Options such as calcium channel blockers and angiotensin-converting enzyme inhibitors (ACEIs) may be preferable in this context. Reducing SBP while stabilizing PP fluctuations may become a potentially effective postoperative BP management strategy. In the future, prospective studies are needed to determine whether PP is a modifiable therapeutic target or a prognostic marker.

## Limitation and expectation

Among 12 postoperative variables, we identified mean PP as an effective predictor of poor prognosis and mortality in patients with AIS-LVO after thrombectomy; this finding was confirmed through multiple statistical methods. However, this study had some limitations. First, the retrospective observational design may have introduced residual confounding due to unmeasured variables not considered in the analyses. Second, attending physicians typically choose antihypertensive medications based on guidelines and clinical experience, leading to variability in drug selection, dosage, and timing of intervention. To address these issues, future studies should collect detailed data regarding post-EVT medication use to better adjust for these confounding factors. Third, .unlike the well-established management strategies for SBP or MAP, no medications are currently available to directly regulate PP, even in intensive care unit (ICU) settings. However, our findings suggest that higher mean PP within 24 h post-EVT is related to higher risk of sICH, unfavorable functional outcomes, and mortality in stroke patients with AIS-LVO, which may be beneficial to explain the neutral or negative outcomes observed in previous RCTs on blood pressure control. Therefore, although PP cannot be directly controlled, medications that lower SBP with minimal impact on DBP could help avoid substantial increases in PP. Options such as calcium channel blockers and angiotensin-converting enzyme inhibitors (ACEIs) may be preferable in this context. Despite the superior associations and diagnostic efficiency of PP compared with SBP or DBP in our study, PP may function as an indirect marker rather than an actionable parameter. Fourth, in the ICU, patients’ BP was monitored by trained senior caregivers using noninvasive oscillometric devices to minimize variability. This method is simple, widely applicable, and nontraumatic, but the inherent mechanics of oscillometric devices are primarily accurate for MAP. SBP and DBP are calculated using proprietary algorithms, thus introducing potential inaccuracies in mean PP measurements. Thus far, no statistically significant differences between invasive and noninvasive BP measurements have been reported in the literature. Noninvasive methods are more widely applicable and practical for the postoperative management of stroke patients. Future studies should focus on developing more direct and objective methods for PP assessment after EVT to further elucidate the impact of mean PP on prognosis in patients with AIS-LVO. Finally, We did not collect information on the types, dosages, and durations of intravenous antihypertensive drugs, and thus were unable to know how the use of IV antihypertensives differed between the favorable and unfavorable outcome groups or weather IV antihypertensive use could influence post-EVT PP values. Due to the complex effects of different antihypertensive drugs on BP, further exploration of the types and dosages of antihypertensive drugs used is needed in the future, with the aim of providing more insights for subsequent RCT studies. Further research is needed to validate our findings and address this limitation.

## Conclusions

Mean PP within 24 h after EVT was independently and linearly associated with poor functional outcomes, sICH, and short-term mortality. A mean PP below approximately 57 mmHg was associated with better outcomes, particularly in patients under 80 years, those undergoing multiple thrombectomy passes, and those with anterior circulation infarctions. These findings suggest that SBP reduction and stabilization of PP may be important components of BP management in patients with AIS-LVO. Further prospective studies are needed to determine whether PP is a modifiable therapeutic target or a prognostic marker.

## Electronic supplementary material

Below is the link to the electronic supplementary material.

Supplementary Material 1

Supplementary Material 2

## Data Availability

Data and material are not publicly available, but can be requested through contacting Dr. Li Jianru.
