# Supplementary material for: Pulse pressure after thrombectomy predicts functional outcomes and mortality in acute ischemic stroke with large artery occlusion
Source: Sci Rep. 2025 Aug 12;15:29448. doi: 10.1038/s41598-025-12962-z (PMC12340056; doi:10.1038/s41598-025-12962-z)
Supplement: Supplementary file 1 — Supplementary Material 1 [file 41598_2025_12962_MOESM1_ESM.docx]

**Online supplementary figure 1. Column chart and heat map display of missing values of variables in the queue of patients with AIS-LVO.**


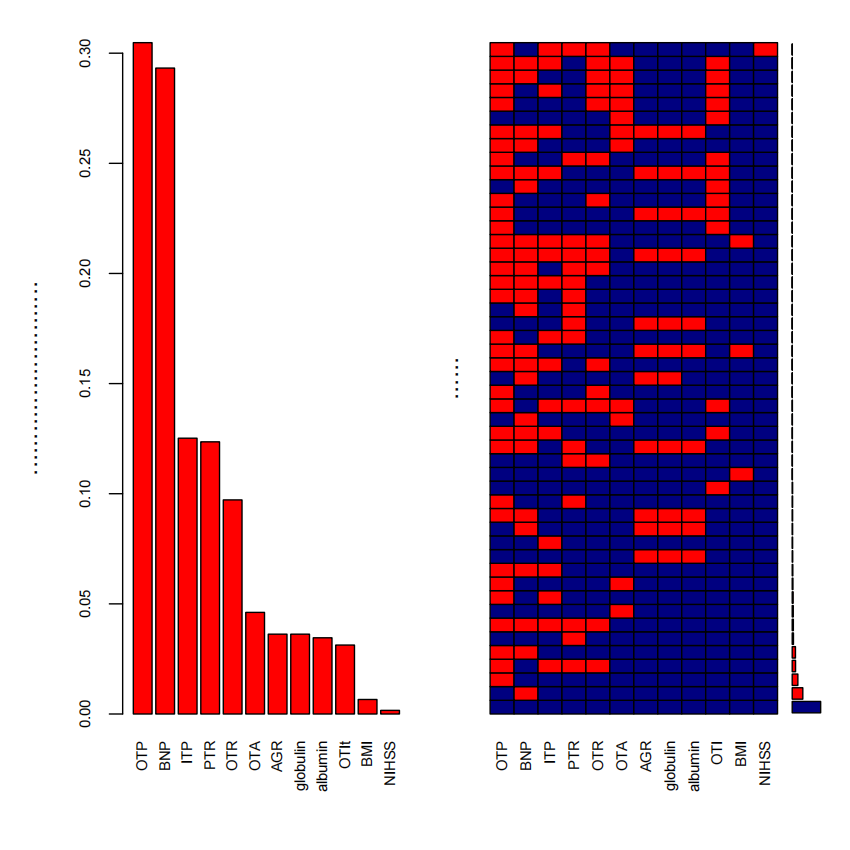


**Online supplementary figure 2. The diagnostic efficacy of postoperative blood pressure variables (including mean SBP, mean PP, maximum SBP and SBP-DMM) on unfavorable prognosis.**


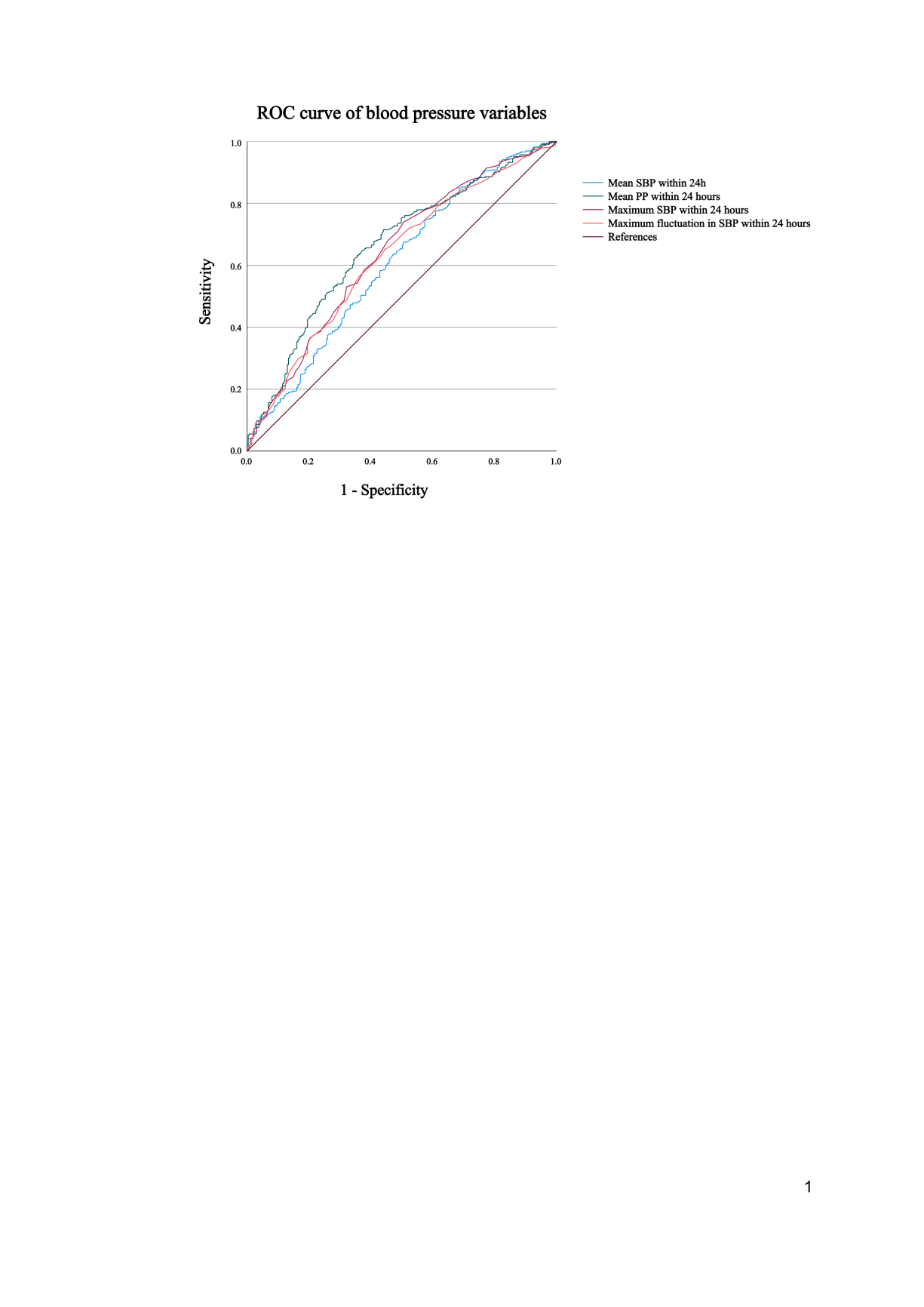


**Online supplementary figure 3. The comparison of blood pressure parameters within 24 hours after EVT for patients with good prognosis and those with poor prognosis.**


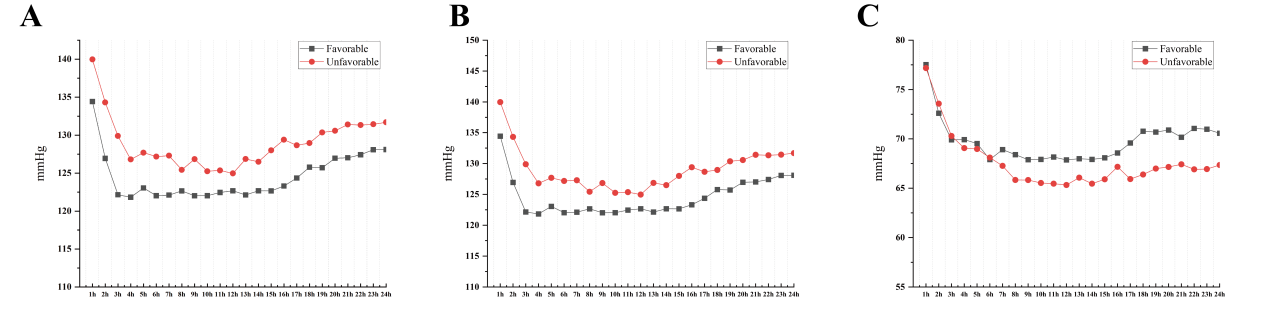


3A. The trend curves of PP changes within 24 hours after EVT for patients with good prognosis and those with poor prognosis. 3B. The trend curves of SBP changes within 24 hours after EVT for patients with good prognosis and those with poor prognosis. 3C. The trend curves of DBP changes within 24 hours after EVT for patients with good prognosis and those with poor prognosis.
